# Simple Continued Fever

**Published:** 1881-04

**Authors:** 


Simple Continued Fever.—

R. Acid hydrobrom ........................ʒj.

Syr. simplicis.........................ʒii.

Aq.....................................℥j.

M. Sig.—Every hour.—*Fothergill.*

Dr. Fothergill, in speaking of the above formula, says it will probably constitute *par excellence* the fever mixture of the future. It is especially indicated where there is cerebral disturbance.—*Indiana Medical Reporter.*

